# High Integrity and Fidelity of Long-Term Cryopreserved Umbilical Cord Blood for Transplantation

**DOI:** 10.3390/jcm10020293

**Published:** 2021-01-14

**Authors:** Gee-Hye Kim, Jihye Kwak, Sung Hee Kim, Hee Jung Kim, Hye Kyung Hong, Hye Jin Jin, Soo Jin Choi, Wonil Oh, Soyoun Um

**Affiliations:** 1 Biomedical Research Institute, MEDIPOST Co., Ltd., Seongnam-si, Gyeonggi-do 13494, Korea; haha38@medi-post.co.kr (G.-H.K.); jihyek@medi-post.co.kr (J.K.); genny77@medi-post.co.kr (H.J.J.); sjchoi@medi-post.co.kr (S.J.C.); 2 Cord Blood Bank, MEDIPOST Co., Ltd., Seongnam-si, Gyeonggi-do 13494, Korea; sswan823@medi-post.co.kr (S.H.K.); special@medi-post.co.kr (H.J.K.); genetichong@medi-post.co.kr (H.K.H.)

**Keywords:** cord blood, long-term cryopreservation, total nucleated cells, recovery

## Abstract

Umbilical cord blood (UCB) is used as a source of donor cells for hematopoietic stem cell (HSC) transplantation. The success of transplantation is dependent on the quality of cord blood (CB) units for maximizing the chance of engraftment. Improved outcomes following transplantation are associated with certain factors of cryopreserved CB units: total volume and total nucleated cell (TNC) count, mononuclear cell (MNC) count, and CD34+ cell count. The role of the storage period of CB units in determining the viability and counts of cells is less clear and is related to the quality of cryopreserved CB units. Herein, we demonstrate the recovery of viable TNCs and CD34+ cells, as well as the MNC viability in 20-year-old cryopreserved CB units in a CB bank (MEDIPOST Co., Ltd., Seongnam-si, Gyeonggi-do, Korea). In addition, cell populations in CB units were evaluated for future clinical applications. The stable recovery rate of the viability of cryopreserved CB that had been stored for up to 20 years suggested the possibility of uses of the long-term cryopreservation of CB units. Similar relationships were observed in the recovery of TNCs and CD34+ cells in units of cryopreserved and fresh CB. The high-viability recovery of long-term cryopreserved CB suggests that successful hematopoietic stem cell (HSC) transplantation and other clinical applications, which are suitable for treating incurable diseases, may be performed regardless of long-term storage.

## 1. Introduction

Cell therapy, which plays a central role in regenerative medicine, involves the application of viable cells for treating or curing medical conditions or diseases. Umbilical cord blood (UCB) is a primitive and alternative source of hematopoietic stem cells (HSCs) and serves as a promising and established curative therapeutic agent for hematological malignancies and life-threatening conditions, primarily hematological and immune system-related genetic disorders [1,2,3]. Viable cells have a short shelf-life at ambient temperatures, which complicates their storage and delivery to patients. To overcome this shortcoming of cell therapy, cell and tissue preservation in cord blood (CB) banks is practiced to preserve living cells under artificial conditions [4]. Cryopreservation, which is a process for preserving cells and tissues at significantly low temperatures, is currently the only method that allows the long-term storage of living cells and tissues for several years and up to decades [5]. It is important to retain the viability of frozen cells by maintaining ultralow temperatures with stable conditions for as long as possible [6,7].

CB banking is increasingly performed for improving chances of potential future transplantation. According to previous reports, UCB contains approximately 10% less stem cells than bone marrow [8,9], but UCB contains similar numbers of progenitor cells (CD34+ cells) volume per volume with bone marrow. However, CB transplantation does not require an exact genetic match. Since the first successful transplantation of cord blood in 1988, quality issues in cord blood specimens have been addressed [10,11]. Therefore, the maintenance of the quality and viability of infused total nucleated cells (TNCs) or CD34+ cells is critical for successful transplantation, owing to retaining adequate cell numbers for future transplantation [12]. Typically, cord blood is processed by depleting red blood cells (RBCs) and plasma. After post-processing with programmable controlled freezing, the CB unit is transferred to a liquid nitrogen tank and stored at −196 °C. A previous report demonstrated over 90% recovery of hematopoietic progenitor cells from cryopreserved CB stored for more than 10–15 years [13].

In this study, we evaluated whether CB banking helps retain the viability of cryopreserved cells stored for up to 20 years. In addition to an efficient storage state, the ability to freeze and retrieve UCB indicates the recovery rate of functional responsive lymphocytes for future therapeutic applications.

## 2. Experimental Section

### 2.1. Collection, Cryopreservation, and Separation of UCB Cells

Human UCB, obtained during deliveries, was collected in blood bags containing 24.5 mL of citrate phosphate dextrose adenine (CPDA-1) as an anti-coagulant, as described earlier [14]. The study was conducted in accordance with the Declaration of Helsinki, and the protocol was approved by the Korean Public Institutional Review Board (P01-202002-31-001). The UCB collection procedure was continued after obtaining informed consent from donors. During a processing step, RBCs and plasma were separated from the specimens and depleted according to a method reported earlier [15]. After depletion of RBC and plasma, 20 mL of concentrated leukocyte-rich plasma was obtained and added to a freezing bag (ThermoGenesis Corp, Rancho Condova, CA, USA) containing 5 mL of 10% cryoprotectant (DMSO/dextran). CB (contained in the bag) was subjected to a controlled- rate freezing (CRF) procedure and stored at −196 °C in a liquid nitrogen tank (MVE Storage Tank, Chart-MVE, Brentwood, NH, USA). To determine the stability of TNCs, the viable cells were analyzed after processing and before cryopreservation.

For the evaluation of long-term storage stability, three sets of CB units were analyzed. In the first set, a total of 87 CB units deposited between 2000 and 2011 at MEDIPOST Co., Ltd. and 11 fresh UCB units donated in 2020 were analyzed. Among the cryopreserved CB units, only the disqualified units owing to insufficient TNC counts were used for the experiments (<7 × 10^8^ cells/unit of TNC), based on the guidelines of the Korea National Laws on Cord Blood Management and Research (2011). In the second set, 116 CB units transplanted between 2001 and 2016 were analyzed. The segments attached to the freezing bags for the 116 CB units were used to assess the viability and TNC cell count recovery before transplantation. In the third set, for long-term tracing, 40 CB units donated from 2009 to 2018 were used. For assessing the long-term storage stability, the viability, TNC count, and CD34+ cells count were evaluated every year for 1–10 years. Each CB unit was divided into multiple segments. Each vial (total 215) was analyzed annually after thawing.

### 2.2. Hematological Cell Counts and Enumeration of CD34+ Cells

The TNC count was determined before and after cryopreservation using a hematology analyzer (Siemens ADVIA2120 and ADVIA 2120i, Malvern, PA, USA). CD34+ cells were quantified using a stem cell enumeration kit (BD Biosciences, Franklin Lakes, NJ, USA) according to the manufacturer’s instructions. CD45-fluorescein isothiocyanate (FITC) and CD34-phycoerythrin (PE) antibodies (BD Biosciences, PharMingen, San Jose, CA, USA) were used to detect the leucocytes and hematopoietic stem/precursor cells, respectively. 7-Aminoactinomycin (7-AAD, BD Bioscience), a nucleic acid dye, was used for evaluating cell viability. Flow cytometry was performed using a flow cytometer (before processing: BD FACSCalibur, Beckton Dickinson, San Jose, CA, USA and after processing: MACSQuant, Miltenyi Biotec, Bergisch Gladbach, North Rhine-Westphalia, Germany).

### 2.3. Cell Viability Evaluation

Trypan blue dye-exclusion was performed to measure the viability of mononuclear cells (MNCs). An Annexin V-FITC early apoptosis detection kit (Cell Signaling, Boston, MA, USA) was used to evaluate cell viability and detect apoptosis according to the suggested protocol. The stained cells were analyzed using a flow cytometer (MACSQuant).

### 2.4. Frequencies of Cell Types in UCB

To analyze the cell populations and types, MNCs were isolated using Ficoll-Hypaque solution (GE Healthcare, Uppsala, Sweden) according to the suggested protocol. The MNCs were washed and resuspended with phosphate buffer saline (PBS) to assess and analyze the surface markers. The cells were incubated with human CD34, CD14, and CD3-phycoerythrin (PE) antibodies and stained with CD19-fluorescein isothiocyanate (FITC), CD56-Alexa 488, and CD45-allophycocyanin (BD Biosciences). Isotype controls were matched to detect nonspecific background signals corresponding to negative controls. The cells were analyzed using a flow cytometer (MACSQuant).

### 2.5. Statistical Analysis

Data are presented as mean ± standard deviation (SD). Statistical analysis involved multiple comparisons using one-way analysis of variance (ANOVA), followed by Bonferroni’s multiple comparisons test using Prism 7 software (GraphPad, San Diego, CA, USA). A *p* < 0.05 was considered to represent a statistically significant difference.

## 3. Results

### 3.1. Recovery of Viability, TNCs, and Total CD34+ Cells from Cryopreserved UCB

**Figure 1:**
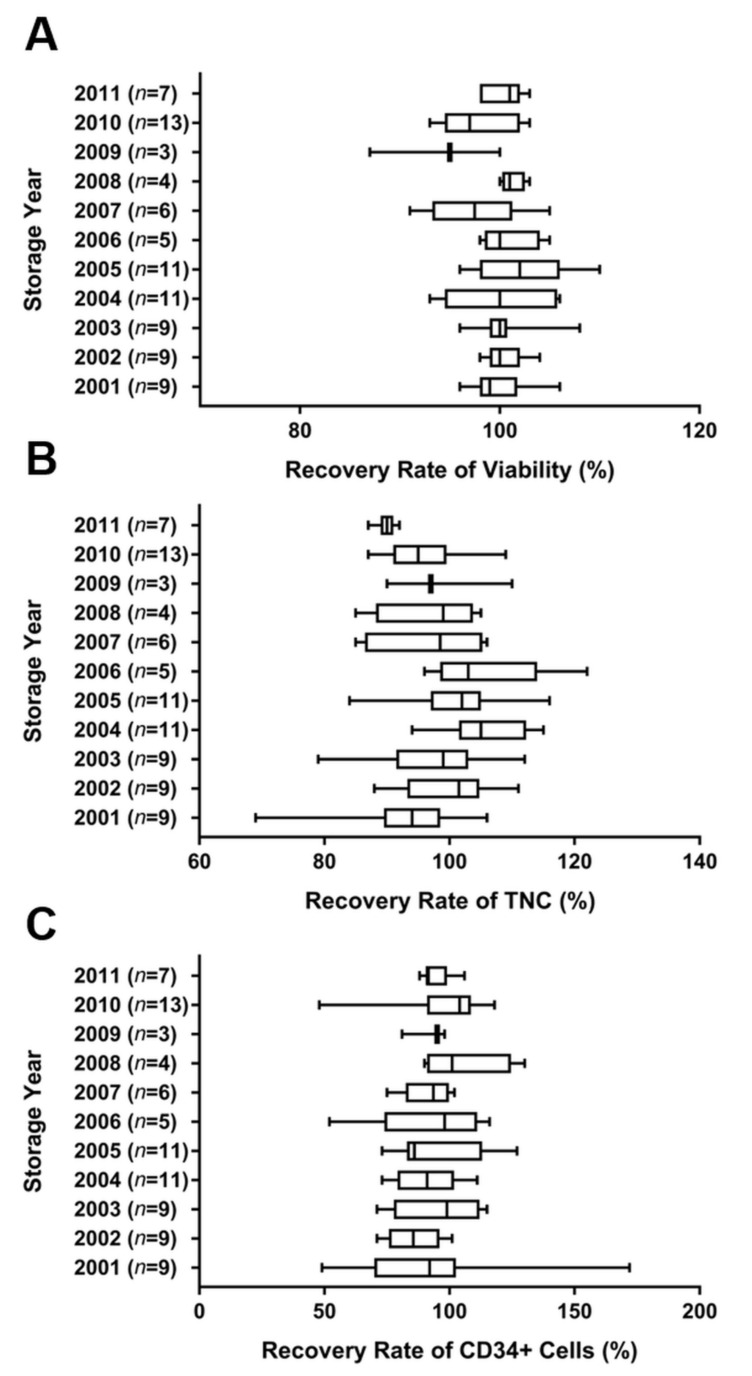
Recovery rates of viability (**A**), TNCs (**B**), and total CD34+ cells (**C**) from 87 cryopreserved human CB units stored from 2001 to 2011. The viability and the cell types were analyzed before and after cryopreservation. The average recovery rates of viability, TNC count, and total CD34+ cell count in total cryopreserved CB were 99.8 ± 4.0%, 98.2 ± 8.7%, and 93.8 ± 18.0%, respectively. Values of *p* > 0.99 (**A**), *p* > 0.06 (**B**), and *p* > 0.99 (**C**) indicated no significant difference between samples with different cryopreservation periods. Data are presented as mean ± standard deviation of mean. Abbreviations: CB, cord blood; TNCs, total nucleated cells.

To evaluate the phenotype of cryopreserved CB, the number of TNCs, total CD34+ cells, and viable cells were determined before and after cryopreservation. The cryopreserved CB units remained stored from 2001 to 2011. The storage periods for the CB units ranged from 10 to 20 years. Eighty-seven units were used for the analysis of viability, TNC count, and total CD34+ cell count before and after cryopreservation. Viability recovery rate was observed in 99.8 ± 4.0% of the CB units. There was no significant difference between the units based on the storage periods. The average cell viability rate before and after cryopreservation was 87.8 ± 3.5% and 87.6 ± 4.0%, respectively (*p* > 0.99). The average TNCs count was 6.1 ± 0.6 (×108/unit) at storage and 6.1 ± 0.7 (× 108 cells/unit) after thawing. The average total CD34+ cell counts ranged from 0.9 to 6.4 (× 106 cells/unit) at storage, and the average total CD34+ cells ranged from 0.8 to 5.7 (× 106 cells/unit) after thawing. The average recovery rate of TNCs in all the CB units was 97.9 ± 9.4% (*p* > 0.06), while the average recovery rate of CD34+ cells was 93.8 ± 18.0% (*p* > 0.99). The recovery rates of TNCs and total CD34+ cells did not differ significantly between CB units with different storage periods (Figure 1).

### 3.2. Long-Term Storage Stability (from 1 to 10 Years)

Data for the counts of viable cells, TNCs, and CD34+ cells in the CB units were recorded between 1 and 10 years. Long-term storage efficacy was evaluated by tracing the number of viable cells, TNCs, and CD34+ cells for the 10 years. The donated CB units (*n* = 40) were stored, and the hematological and CD34+ cell counts were determined annually using a flow cytometer. The average recovery rates of viable cells, TNCs, and CD34+ cells in total CB units were 96.6 ± 4.0%, 86.2 ± 7.0%, and 82.0 ± 19.8%, respectively. The recovery rates of viable cells, TNCs, and CD34+ cells were not affected significantly by the storage period, as observed when the segment was inspected annually (Table 1).

### 3.3. Population of MNCs Isolated in Cryopreserved and Fresh UCB

Cryopreserved and fresh CB units were subjected to flow cytometric analysis, and a scatter plot was generated. After RBC depletion, whole CB cells were represented on a scatter plot that revealed the presence of three major components: lymphocytes, monocytes, and granulocytes. The three distinct cell populations are indicated with green (granulocytes), blue (monocytes), and red (lymphocytes) (Figure S1A,C). MNCs from the cryopreserved and fresh CB samples showed the depletion of granulocytes after Ficoll separation (Figure S1B,D). After RBC depletion and Ficoll separation, cell populations in MNCs from cryopreserved CB revealed 8.5 ± 8.2% of monocytes and 93.0 ± 4.7% of lymphocytes populations. The percentages of monocytes and lymphocytes in MNCs isolated from fresh CB were 8.6 ± 4.3% and 98.0 ± 2.3%, respectively. There was no significant difference in the cell populations between cryopreserved and fresh CB samples (Figure S1, Table 2).

Cell populations in MNCs isolated from CB were analyzed using antibodies. A comparison of the six types is illustrated in Table 2. The mean percentage of each cell type among the leukocytes in CB units (total 66 lots) cryopreserved from 2000 to 2011 was indicated and compared with leukocytes from fresh CB samples (total 6 lots). The results included the stem cell population in MNCs from cryopreserved CB, including that of CD34+ HSCs (0.5 ± 0.4%) and immune cell populations, such as CD3+ T lymphocytes (46.4 ± 15.5%), CD19+ B lymphocytes (0.8 ± 0.8%), and CD56+ natural killer cells (0.3 ± 0.6%). Cell populations in MNCs from fresh CB revealed CD3+ T lymphocytes (56.8 ± 10.0%), CD19+ B lymphocytes (1.5 ± 0.4%), and CD56+ natural killer cells (0.7 ± 0.4%). There was no significant difference between the mean percentages of each cell type in cryopreserved and fresh CB units (*p* > 0.99).

### 3.4. Comparison of MNC Viability in Cryopreserved and Fresh UCB

**Figure 2:**
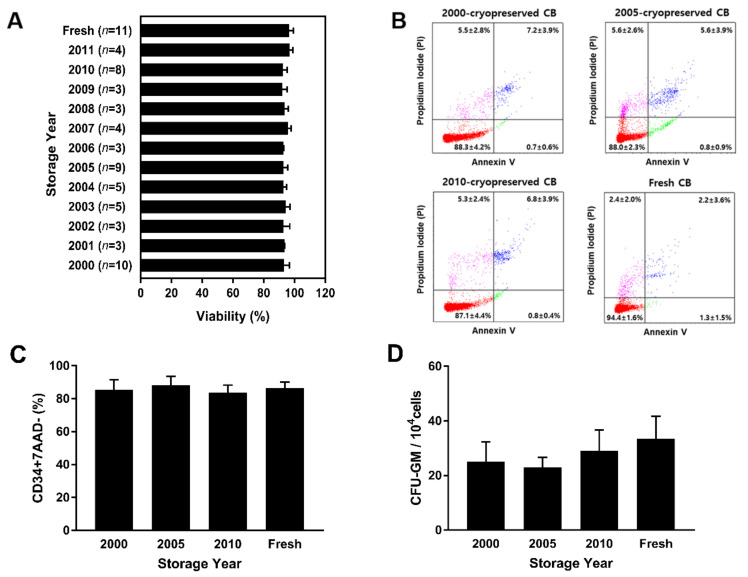
Comparison of MNC viability in long-term cryopreserved and fresh umbilical CB samples by Annexin V/propidium iodide staining. (**A**) After RBC depletion and Ficoll separation, MNC viability was assessed in 60 cryopreserved CB units (stored from 2000 to 2011) and 11 fresh CB units. There was no significant difference between MNC viabilities in cryopreserved and fresh CB units (*p* > 0.99). The average viability percentage was 93.6 ± 1.4%. (**B**) Annexin V/propidium iodide staining revealed that there was no correlation between cryopreservation period and cell viability. (**C**) The viability of CD34+ cells in cryopreserved (3 units stored from 2000, 2005, and 2010) and fresh CB (3 units) was analyzed by flow cytometry with 7-AAD staining. (**D**) In vitro CFU-GM assay provided the colony number of granulocyte-macrophage progenitor cells for 14-day culture period (*p* = 0.32). Data are presented as mean ± standard deviation of mean. Abbreviations: CB, cord blood; MNCs, mononuclear cells; 7-AAD, 7-Aminoactinomycin; CFU-GM: colony-forming unit-granulocyte, macrophage.

MNCs, a mixture of angiogenic stem and progenitor cells, non-hematopoietic cells, and hematopoietic cells were separated using Ficoll density gradient centrifugation. The viability of MNCs isolated from long-term cryopreserved CB stored from 2000 to 2011 was compared to that of MNCs isolated from fresh CB. After separation using Ficoll, the mean percentages of viable MNCs isolated from cryopreserved CB and fresh CB were 93.6 ± 1.4% and 96.4 ± 2.8%, respectively. There was no significant difference between the mean percentage of viability of MNCs isolated from cryopreserved and fresh CB (*p* > 0.99). The results show that the viability of MNCs remained unaffected by the storage period (Figure 2A), as indicated by the levels of apoptosis determined using Annexin V/propidium iodide double staining performed using MNCs isolated from cryopreserved CB units (2000, 2005, and 2010) and fresh CB units. The assessment of MNC viability in 2000, 2005, and 2010 indicated that the viable MNC count did not differ significantly in CB cryopreserved for different periods. MNCs isolated from samples cryopreserved in 2000, 2005, and 2010 showed a mean viability of 88.3 ± 4.2%, 88.0 ± 2.3%, and 87.1 ± 4.4%, respectively, whereas those isolated from fresh CBs showed a mean viability of 94.4 ± 1.6%. The overall proportions of dead, necrotic, and apoptotic cells among cryopreserved MNCs did not differ significantly from those among fresh MNCs (Figure 2B). The viability of CD34+ cells on total nucleated cells was observed with 7-AAD staining. Average viability of CD34+ cells isolated from CB cryopreserved in 2000 (85.6 ± 6.1%), 2005 (88.3 ± 5.2%), and 2010 (83.7 ± 4.5%), and from fresh CB (86.4 ± 3.8%) was 86.0 ± 4.9%. There was no significant difference in the viability of CD34+ cells from cryopreserved and fresh CB (Figure 2C). There was no significant change in MNCs and CD34+ cell viability and apoptosis in CB units stored for over 20 years. To confirm that long-term cryopreserved cells were still functionally viable, a colony forming unit (CFU) assay was performed using MNCs isolated from CB stored in 2000, 2005, and 2010. Compared to MNCs isolated from fresh CB, the number of CFU-GM (granulocyte-macrophage) had no significant difference (*p* = 0.32). Overall, the results show that long-term storage CB units had no significant difference on the functional viability and potency of MNCs and CD34+ cells.

### 3.5. Characteristics of Store CB Units Used for Transplantation

Table 3 summarizes data on the cryopreservation period (time interval between donation and transplantation), TNC count, total CD34+ cells, and viability of CB units. To determine the impact of cryopreservation on TNC counts and CD34+ cells, TNC counts and CD34+ cells before and after cryopreservation of CB units used for transplantation were determined. For HSC transplantation, 3.8 ± 2.9 years of cryopreservation period were considered. Overall, 557 CB units used for transplantation showed a mean viability of 83.2 ± 5.7% at transplantation. The TNC counts were 9.8 ± 3.9 (× 10^8^ cells/unit) at the storage year and 9.8 ± 4.0 (× 10^8^ cells/unit) at the transplantation year. These results indicate that the cryopreservation period did not affect the viability and TNC counts (Table 3).

## 4. Discussion

To date, CB has been used as a source of hematopoietic stem and progenitor cells in over 40,000 clinical hematopoietic cell transplantations to treat hematological, metabolic, immunological, neoplastic, and neurological disorders. Approximately 5 million units of CB are stored in public and private cord banks worldwide [4]. This study presented an opportunity to assess the quality of cryopreserved CB units stored in private Korean cord banks since 2000. Owing to the requirements for rigorous processing and cryopreservation before transplantation, the final quality of long-term cryopreserved CB units remains uncertain. The study aimed to assess critical factors for CB recovery rates, including viability, TNC count, and CD34+ cell count in the hematopoietic stem cell and immune cell populations before and after cryopreservation.

The total number of nucleated cells transplanted strongly correlates with clinical outcomes. On average, a typical viable CB unit has a volume of 120 mL and contains 0.8–3 × 10^9^ TNCs. After processing, a loss of 10–20% of initially harvested blood was indicated. Generally, the minimum requirement for transplantation is 2.5 × 10^7^ TNCs per kg of recipient body weight [16,17]. The success of transplantation depends completely on the quality of cryopreserved CB units maintained by the cord bank [18]. To improve the quality of cryopreservation in CB storage, requalification testing prior to the release of a unit utilizing a contiguous segment of CB samples is being developed as a method to confirm unit identity and the growth of hematopoietic progenitor cells before transplantation [11,19,20]. In cord blood transplantations, the viability of stem cells is associated with the recovery of patients. The CB processing method is critical for maintaining the most viable stem cells. In this research, the Rubinstein method was used to collect and process plasma-depleted CB for clinical transplantation [15]. It is important to establish how long CB can be maintained viably in cryogenic storage

Correlation between the duration of storage and transplant outcomes has not been reported. CB units that have been stored for over 10 years have not been used successfully in transplants. There is no consensus regarding the shelf life of CB units, even when stored under suitable conditions in liquid nitrogen. Previously, it was reported that CB units could be stored for at least 23.5 years with minimal HSC loss [13,21,22]. Herein, we demonstrated the highly efficient recovery of viable cells, TNCs, and CD34+ cells from stored CB. Usually, the hematopoietic potential of CB is estimated by the number of cells expressing the CD34 antigen with high functionality. The use of cryopreserved CB in recent times has expanded its range of applications in transplantation and regenerative medicine. Monocytes isolated from cryopreserved CB could be potentially transplanted for treating inherited demyelinating conditions of the central nervous system [23]. Additionally, dendritic cells derived from cryopreserved CB monocytes were observed to exhibit antitumor potential [24]. Induced pluripotent stem cells were generated from HSCs isolated from 21- to 23.5-year-old cryopreserved CB samples that showed optimal storage recovery of HSCs for future clinical utility [22,25]. In addition, the utility of non-manipulated cryopreserved CB in the treatment of type 1 diabetes and acquired neurological injuries has been assessed in clinical studies [26,27]. 

Several clinical applications have been determined for over 500 cryopreserved CB units released from private CB banks. In Korea, there are 17 public and private cord blood banks storing more than 500,000 CB units currently. MEDIPOST Co., Ltd. private cord blood bank, the first private bank in Korea, was established in 2000, and 254,505 units were cryopreserved until December 2020. Until now, 557 CB units were used for either autologous or allogeneic transplants (Table 4). The longest storage period of a CB unit used for transplantation was 17 years. A CB unit stored from 2003 was used for allogenic transplantation in 2020 for a patient with acute myeloid leukemia (Table 3 and Table 4). In this study, we analyzed 128 CB units among 557 units that were used for transplantation and have been cryopreserved for >10 years. Our study demonstrated that the 557 transplanted CB units had a higher viability rate and a consistently higher recovery rate of TNCs and CD34+ cells. A large percentage of cryopreserved CB units have been used in clinical applications, such as for the treatment of brain injury, developmental disorders, HSC transplant indication, acquired hearing loss, and autoimmune disorders. Several studies have been conducted to improve outcomes following CB transplantation with minimal manipulations [28]. Our study provided data and compared the cell populations in CB units that had been cryopreserved for 20 years with those in fresh CB units. The findings of this study support the use of CB units that have undergone long-term cryopreservation and should reassure clinicians regarding the potential of CB transplantation in other clinical applications besides HSC transplantation.

This study has several limitations. First, clinical results about transplanted CB units were unavailable. Restricted clinical outcomes—including graft-versus-host disease, transplantation-related mortality, relapse, and survival—could not be included in the long-term cryopreservation correlations. In addition, we excluded CB units used in double transplantation to minimize confounding the results. Finally, only CB units with a TNC of 8.0 × 10^8^ cells/unit and over that had been cryopreserved since 2011 were used. Therefore, we did not include the data of cryopreserved CB from 2012 to 2019. CB units with TNCs of under 8.0 × 10^8^ cells/unit were not allowed to cryopreserve.

The clinical outcomes of transplantation are influenced by the number of TNCs and CD34+ cells, and by HLA (human leukocyte antigen) matching [29,30,31]. However, the reduced CB volume post-processing has not been observed to affect transplantation outcomes [32,33]. Cryopreservation for future use and establishment of insurance reimbursement have met the increasing demand for precision health care. The safety and success of CB transplantation relies on the integrity of cryopreservation conditions. Loss of integrity during long-term storage can lead to failures in CB maintenance and errors in the quality assessment of stored CB units [12,18,20]. The stability of long-term cryopreserved CB has to be based on the satisfactory recovery of TNCs and viable CD34+ cells after long-term storage. In order to evaluate the cryopreservation and storage over time on the stability and quality of CB units, the viability of TNC and CD34+ cells of each segment from cryopreserved CB units were analyzed annually in this study. With high CD34+ cells’ viability and a consistently high recovery rate of TNCs, the storage process in a bank is stabilized, reliably controlling the quality of thawed cells. We also confirmed the functional viability and potency of MNCs and CD34+ cells with an evaluation by CFU-GM assay. In this study, we demonstrated that the duration of cryopreservation did not significantly affect the recovery of viability, TNCs, and CD34+ cells, which is vital for successful transplantation. In clinical practice, the use of CB units stored for more than 10 years is avoided in general cases and transplantations [34,35]. The findings of this study are critical in this context, as they provide evidence that the long-term cryopreservation of CB is not detrimental to the outcomes of recovery of viable cells and TNCs.

The limitation of HSC transplantation is the reduced viability and poor cell quality after thawing of specimens with a long cryopreservation period. However, our study demonstrated that the quality of cryopreserved CB units did not correlate with the cryopreservation period. Although the cryopreservation and thawing processes may damage cord blood cells, the cell loss is less likely to affect the viability and recovery of CD34+ cells and TNCs. Furthermore, long-term cryopreservation did not affect the immune cell population, which could be utilized in future clinical applications. These results support the use of CB units that have undergone long-term cryopreservation and provide assuring evidence in favor of the clinical applicability of cryopreserved CB.

## Figures and Tables

**Table 1:** Tracing of recovery rate of viability, TNCs, and CD34+ cells from 40 cryopreserved human umbilical cord blood units by checking CBs annually for 10 years (AVE ± SD, %).

Storage Duration (Years)	Total Check Point (Annual)	Recovery Rate of Viability (%)	Recovery Rate of TNCs (%)	Recovery Rate of CD34+ Cells (%)
10	3	94.6 ± 1.0	80.5 ± 2.7	66.0 ± 14.9
9	7	93.4 ± 3.3	80.9 ± 4.6	81.0 ± 19.4
8	12	96.5 ± 4.3	80.7 ± 4.4	80.8 ± 22.9
7	16	96.2 ± 4.6	82.3 ± 4.3	82.7 ± 18.7
6	20	96.6 ± 4.1	84.7 ± 5.1	83.9 ± 18.0
5	24	96.2 ± 4.0	86.8 ± 4.9	78.0 ± 22.2
4	28	96.7 ± 3.5	85.9 ± 9.4	80.2 ± 20.6
3	32	96.8 ± 3.6	87.0 ± 7.2	81.9 ± 23.5
2	36	97.5 ± 3.6	87.9 ± 6.7	82.5 ± 17.7
1	37	96.7 ± 4.9	89.4 ± 6.9	86.0 ± 17.4
Total	215	96.6 ± 4.0	86.2 ± 7.0	82.0 ± 19.8

Abbreviations: AVE, average; SD, standard deviation; TNCs, total nucleated cells.

**Table 2:** Cell populations in MNCs isolated from 20-year cryopreserved and fresh umbilical cord blood (AVE ± SD, %).

Storage Year	*n*	CD45+ Lymphocytes	CD14+ Monocyte	CD34+ HSCs	CD45+/CD3+ T Cells	CD45+/CD19+ B Cells	CD56+ Im/m NK Cells
2000	10	90.8 ± 3.6	9.0 ±5.7	0.6 ± 0.5	46.2 ± 16.8	0.3 ± 0.4	0.3 ± 0.3
2001	5	95.1 ± 1.4	11.8 ± 15.8	0.4 ± 0.4	47.5 ± 11.1	1.3 ± 0.9	0.4 ± 0.1
2002	5	93.1 ± 3.9	10.6 ± 4.1	0.8 ± 1.0	37.1 ± 13.5	1.5 ± 1.6	0.4 ± 0.3
2003	4	92.9 ± 5.6	3.6 ± 2.0	0.4 ± 0.4	37.9 ± 16.2	1.7 ± 1.2	0.4 ± 0.3
2004	4	93.3 ± 5.8	8.0 ± 11.1	0.3 ± 0.2	36.7 ± 5.7	0.3 ± 0.2	0.1 ± 0.1
2005	8	90.6 ± 6.2	5.8 ± 4.8	0.4 ± 0.3	51.0 ± 12.7	0.4 ± 0.2	0.3 ± 0.4
2006	4	92.1 ± 6.2	5.0 ± 0.8	0.5 ± 0.2	60.5 ± 22.9	1.0 ± 1.2	1.3 ± 2.1
2007	4	94.3 ± 5.6	5.2 ± 4.5	0.2 ± 0.1	65.3 ± 12.9	1.1 ± 1.0	0.2 ± 0.1
2008	4	94.1 ± 2.3	5.1 ± 5.4	0.6 ± 0.4	35.3 ± 26.5	1.4 ± 1.1	0.1 ± 0.1
2009	4	93.7 ± 2.8	3.8 ± 1.5	0.2 ± 0.2	34.9 ± 14.8	1.2 ± 1.1	0.1 ± 0.1
2010	10	94.5 ± 6.2	12.2 ± 9.8	0.5 ± 0.2	53.9 ± 9.8	0.7 ± 0.6	0.3 ± 0.4
2011	4	94.6 ± 3.2	10.1 ± 12.4	0.2 ± 0.1	38.9 ± 9.3	0.8 ± 0.7	0.2 ± 0.2
Total	66	93.0 ± 4.7	8.5 ± 8.2	0.5 ± 0.4	46.4 ± 15.5	0.8 ± 0.8	0.3 ± 0.6
Fresh	6	98.0 ± 2.3	8.6 ± 4.3	0.6 ± 0.6	56.8 ± 10.0	1.5 ± 0.4	0.7 ± 0.4

Abbreviations: AVE, average; Im/m, immature/mature; MNCs, mononuclear cells; HSCs, hematopoietic stem cells; NK, natural killer; SD, standard deviation.

**Table 3:** Characteristics of 557 patients receiving CB transplantation.

Parameter	Number	Range
**CB unit cryopreservation, yr**	***n***	-
≤5	429	-
5.1 to 10	111	-
10.1 to 15	16	-
15.1 to 21	1	-
**TNC (**×**10^8^/unit)**	**AVE ± SD**	**Range**
Pre-TNC	9.8 ± 3.9	1.1 to 32.4
Post-TNC	9.8 ± 4.0	1.1 to 32.2
**CD34+ cells (**×**10^6^/unit)**	**AVE ± SD**	**Range**
Pre-CD34+ cells	4.1 ± 2.8	0.5 to 23.6
Post-CD34+ cells	3.9 ± 2.7	0.4 to 19.0
**Post-thaw viability (%)**	**AVE ± SD**	**Range**
Viability	83.2 ± 5.7	60.0 to 96.0
**HLA matching**	***n***	-
6/6 or 5/6	441	-
4/6 or less	116	-
**ABO match**	***n***	-
Match	246	-
Minor Mismatch	297	-
Major Mismatch	14	-
**Recipient gender**	***n***	-
Male	336	-
Female	200	-
Unknown	21	-
**Recipient age, year**	***n***	-
≤5	197	-
5.1 to 10	155	-
>10.1	189	-
Unknown	16	-
**Recipient Weight, kg**	***n***	-
≤10	57	-
10.1 to 30	225	-
30.1 to 60	124	-
>60	67	-
Unknown	84	-
**Autologous vs. Allogenic**	***n***	-
Autologous	78	-
Allogenic	479	-
**Diagnosis**	***n***	-
Chronic myeloid leukemia (CML)	18	-
Acute myeloid leukemia (AML)	161	-
Acute lymphoblastic leukemia (ALL)	152	-
Aplastic anemia	26	-
Hemophagocytic lymphohistiocytosis (HLH)	11	-
Cerebral palsy	38	-
Developmental disability	34	-
Others	117	-

Abbreviations: AVE, average; SD, standard deviation; TNCs, total nucleated cells; HLA, human leukocyte antigen; ABO, ABO blood type

**Table 4:** The number of CB units according to the transplantation and storage year.

Storage Year	Cord Blood Transplantation Year																				
	2001	2002	2003	2004	2005	2006	2007	2008	2009	2010	2011	2012	2013	2014	2015	2016	2017	2018	2019	2020	Total
2000	3	2	4	5	4	1	3			1					1						24
2001	10	8	18	14	15	22	7	11	3	5	3	6	2	1							125
2002		1	3	3	5	2	2		2	4	4	1									27
2003			3	22	16	23	10	11	7	7	12	3		2	1		1			1	119
2004				8	18	32	13	7	9	11	2	2	2	3	1						108
2005					1	14	15	7	3	8	1	1	1								51
2006						3	7	1	3	2	1	2	2	1		1					23
2007							3	3	7	7	4	2	2		1						29
2008										3	2				1				1		7
2009											1	3		1				1			6
2010											1		3	3	2	1					10
2011											2	2	1	1	2	1		1			10
2012													2			1		1			4
2013														1	1	2					4
2014															4	1					5
2015																1	1				2
2016																3					3
Total	13	11	28	52	59	97	60	40	34	48	33	22	15	13	14	11	2	3	1	1	557

Empty table indicates no transplantation.

## Data Availability

The data presented in this study are available on request from the corresponding author.

## Supplementary Materials

The following are available online at https://www.mdpi.com/2077-0383/10/2/293/s1. Figure S1: White blood cell populations in total and mononuclear cells isolated from cryopreserved and fresh CB.
