# Supplementary material for: High Integrity and Fidelity of Long-Term Cryopreserved Umbilical Cord Blood for Transplantation
Source: J Clin Med. 2021 Jan 14;10(2):293. doi: 10.3390/jcm10020293 (PMC7830419; doi:10.3390/jcm10020293)
Supplement: Supplementary file 1 [file jcm-10-00293-s001.pdf]

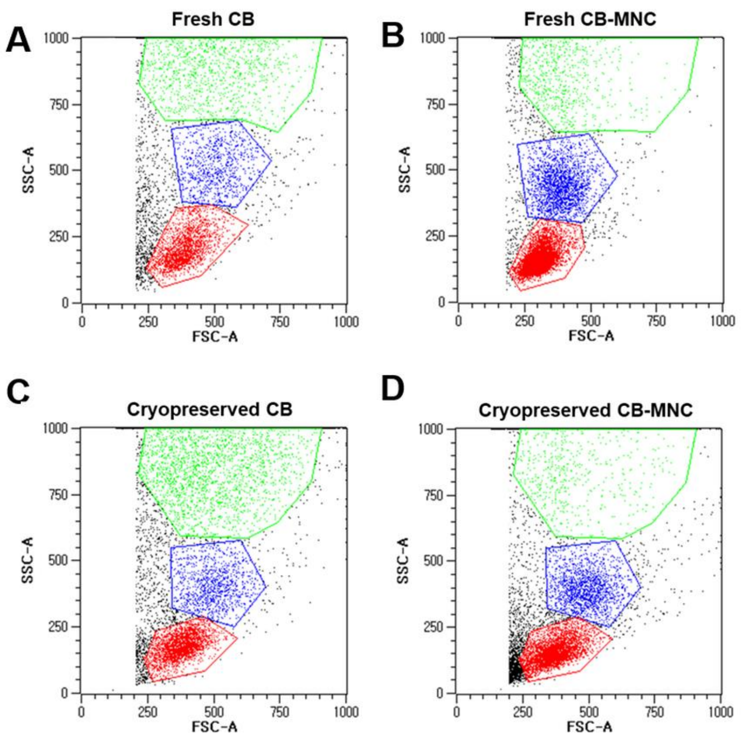

Figure S1. White blood cell populations in total and mononuclear cells isolated from cryopreserved and fresh CB. The three major components of CB indicated in the flow cytometry scatter plot were lymphocytes, monocytes, and granulocytes. The three distinct populations were indicated based on their size and cell complexity parameters, such as granularity. Red, blue, and green color indicated lymphocytes, monocytes, and granulocytes, respectively. (**A, C**) Total CB from cryopreserved and fresh unit CB samples showed the presence of three distinct cell populations. There were no significant differences in the cell populations between fresh and cryopreserved CB units. (**B, D**) After separation of the components of CB using Ficoll, the MNCs were divided into three distinct cell populations. CB, cord blood; MNC, mononuclear cells; SSC, side scatter; FSC, forward scatter.
